# FCRL1 immunoregulation in B cell development and malignancy

**DOI:** 10.3389/fimmu.2023.1251127

**Published:** 2023-09-25

**Authors:** Murali K. Mamidi, Jifeng Huang, Kazuhito Honjo, Ran Li, Edlue M. Tabengwa, Indira Neeli, Nar’asha L. Randall, Manasa V. Ponnuchetty, Marko Radic, Chuen-Miin Leu, Randall S. Davis

**Affiliations:** ^1^Department of Medicine, University of Alabama at Birmingham, Birmingham, AL, United States; ^2^Department of Microbiology, Immunology and Biochemistry, University of Tennessee Health Science Center, Memphis, TN, United States; ^3^Institute of Microbiology and Immunology, National Yang Ming ChiaoTung University, Taipei, Taiwan; ^4^Departments of Microbiology, and Biochemistry & Molecular Genetics, University of Alabama at Birmingham, Birmingham, AL, United States; ^5^O’Neal Comprehensive Cancer Center, University of Alabama at Birmingham, Birmingham, AL, United States

**Keywords:** FCRL1, B cells, lymphocyte development, B cell receptor, signaling, malignancy, immunotherapy

## Abstract

Immunotherapeutic targeting of surface regulatory proteins and pharmacologic inhibition of critical signaling pathways has dramatically shifted our approach to the care of individuals with B cell malignancies. This evolution in therapy reflects the central role of the B cell receptor (BCR) signaling complex and its co-receptors in the pathogenesis of B lineage leukemias and lymphomas. Members of the Fc receptor-like gene family (*FCRL1-6*) encode cell surface receptors with complex tyrosine-based regulation that are preferentially expressed by B cells. Among them, FCRL1 expression peaks on naïve and memory B cells and is unique in terms of its intracellular co-activation potential. Recent studies in human and mouse models indicate that FCRL1 contributes to the formation of the BCR signalosome, modulates B cell signaling, and promotes humoral responses. Progress in understanding its regulatory properties, along with evidence for its over-expression by mature B cell leukemias and lymphomas, collectively imply important yet unmet opportunities for FCRL1 in B cell development and transformation. Here we review recent advances in FCRL1 biology and highlight its emerging significance as a promising biomarker and therapeutic target in B cell lymphoproliferative disorders.

## Introduction

B lymphocytes govern the humoral arm of adaptive immunity and are the sole source of antibody (Ab) production for host defense (1). B lineage development is a carefully regulated stepwise process that begins in the fetal liver and bone marrow (2, 3), and culminates with terminal stages of maturation in secondary lymphoid tissues (4). The unique capacity of B cells to undergo rearrangement and somatic hypermutation of their immunoglobulin (Ig) genes results in remarkable Ab repertoire diversity (5–8). However, the adaptive mechanisms that recombine and modify DNA to provide such broad serologic protection can also drive the development of leukemias and lymphomas, >85% of which derive from B lineage cells (9–12).

Over the past two decades, there has been a growing appreciation for the B cell receptor (BCR) and its surface co-associates in regulating the survival and maintenance of B cells that has also provided new insight into how these components contribute to lymphoproliferative disorders (13–17). Accordingly, multiple surface molecules and signaling proteins that modulate BCR function are targets for therapy in patients afflicted by B cell malignancies (18–21). Breakthrough studies from this evolving field have highlighted the importance of proximal and downstream BCR signaling pathways and identified novel intermediates and genetic mutations that perpetuate pathologic signaling (22–26). A windfall of observations has also uncovered little known factors at the nexus of humoral and pathologic immunity that may advance our understanding of still enigmatic origins and survival mechanisms of B cell lymphoproliferative disorders (27–29).

Members of the Fc receptor–like (*FCRL1-6*) gene family encode type I transmembrane surface glycoproteins preferentially expressed by B cells that possess cytoplasmic tyrosine-based motifs and regulate BCR-mediated signaling responses (30, 31). Among them, FCRL1 representatives are present in both humans and mice, possess intracellular motifs with activating characteristics, and exhibit broad expression on mature B cell populations. Although ligand(s) have not yet been identified, the conserved distribution and signaling features for this receptor interspecies, indicate fundamentally important roles for FCRL1 in immunobiology. Here we review progress in FCRL1 research, including recent systemic studies in mouse knockout models, identification of proximal tyrosine-based recruitment partners, and evidence for possible prognostic roles in human B cell lymphoproliferative disorders and aggressive non-Hodgkin’s lymphomas (NHL). These findings provide fresh insight into this understudied molecule in humoral immunity and highlight the potential for FCRL1 as biomarker and immunotherapeutic target.

## B cell development

After birth, B cells in humans and mice are generated in the bone marrow, with the precursor stages of development dedicated to rearrangement of the IgM heavy chain V, D, and J gene segments (1–3). Expression of a productive IgM heavy chain polypeptide enables formation of the pre-BCR, and serves as an initial regulatory checkpoint prior to initiating V and J gene segment rearrangement at the light chain loci (32, 33). Successful generation and surface expression of immunoglobulin (Ig) heavy and light chain heterodimeric pairs, in association with the cluster of differentiation (CD) 79A (Igα) and CD79B (Igβ) transmembrane components, forms the BCR complex that functions to regulate B cell survival and differentiation (13–15). Pending selection at the immature transitional B cell stage, cells receiving positive signals exit the bone marrow and emigrate to secondary lymphoid tissues, including the spleen, tonsils, and lymph nodes, where they express membrane-bound BCR of both IgM and IgD isotypes (34, 35). In contrast to innate-like marginal zone B cells, that possess a polyreactive Ab repertoire and are geared to rapidly respond to pathogen-associated molecular patterns (36), naïve follicular B cells reside in the mantle zones of lymphoid tissue follicles poised for further antigen-receptor diversification in the germinal center (GC) microenvironment (37, 38). Directed by interactions with T follicular helper cells and follicular dendritic cells, cyclic movement of activated GC B cells between the light and dark zones drives Ig somatic hypermutation, class-switch recombination, and affinity maturation (39, 40). When successful, these carefully regulated developmental steps lead to the terminal differentiation of B lineage cells as Ab-secreting plasma cells or memory B cells that are capable of providing long-term host defense (4, 41). However, the diversification mechanisms that are so critical for generating an extraordinary breadth of antigen receptors to ensure humoral protection are also responsible for pathologic transformation and leukemo/lymphomagenesis (11, 17).

## B cell receptor signaling

During defense responses and antigen engagement, BCR activation is chiefly initiated by Src family tyrosine kinases (e.g., LYN, BLK, FYN) that phosphorylate immunoreceptor tyrosine-based activation motifs (ITAM) in the cytoplasmic tails of the non-covalently associated CD79A and CD79B receptor subunits (42, 43). Phosphorylated CD79A/B ITAMs recruit the spleen-associated tyrosine kinase (SYK) that is crucial for the activation of Src homology 2 (SH2) domain-containing proteins (44, 45). A key target is the B-cell linker protein (BLNK/SLP-65), an adaptor that recruits the Bruton’s tyrosine kinase (BTK) and establishes a platform for integrating multiple intracellular signaling components that propagate downstream cascades (46, 47). These include the mitogen-activated protein kinase (MAPK)/extracellular signal-regulated kinase (ERK) pathway via growth factor receptor-bound protein 2 (GRB2) and the VAV guanine nucleotide exchange factor, nuclear factor of activated T cells (NF-AT) by 1-phosphatidylinositol-4,5-bisphosphate phosphodiesterase gamma-2 (PLCγ2) that facilitates inositol-1, 4, 5-triphosphate (IP_3_) production and calcium signaling, and the nuclear factor kappa-light-chain-enhancer of activated B cells (NF-κB) pathway through diacylglycerol generation and protein kinase C activation (48). A fourth cascade is regulated by the CD19 co-receptor that is the principal effector of phosphoinositide 3-kinase (PI3K) signaling either independently or together with the BCR (49). Following antigen engagement, CD19 and the phosphoinositide 3-kinase adapter protein 1 (PIK3AP1/BCAP), are recruited to the BCR signalosome and tyrosine phosphorylated by LYN and SYK. Activation and recruitment of PI3K (p110δ, p85α) to the membrane leads to the conversion of phosphatidylinositol- (4, 5)-bisphosphate (PIP_2_) producing membrane tethered phosphatidylinositol- (3, 4, 5)-trisphosphate (PIP_3_), which serves as a docking site for the RAC (Rho family)-alpha serine/threonine protein kinase B also known as AKT (50). At the plasma membrane, AKT is a target of modulation by two protein kinases, mammalian target of rapamycin (mTOR) that phosphorylates AKT at a carboxyl-terminal S473 residue and the phosphoinositide-dependent kinase-1 (PDK1) that phosphorylates the activation loop T308 site resulting in full AKT activation (51).

## Pathologic B cell receptor signaling

While transcriptomics have disclosed oncogenic driver mutations, altered gene expression patterns, differential signatures of lymphoma and leukemia subtypes, as well as potential cells of origin, the clinical use of tyrosine kinase inhibitors (TKI) in patients has underscored the complex and pathologic signaling roles orchestrated by the BCR (52–56). With the aid of RNA interference screens and genomics, two general forms of pathological BCR signaling have been identified in lymphoid malignancies (19). Chronic-active BCR signaling mimics the proximal stimulatory properties triggered by antigen-receptor binding. Drivers of this signaling phenotype include somatic mutations that constitutively activate CD79A/B or other downstream intermediates of the NF-kB pathway as well as autoantigens recognized by the BCR of the transformed clone. These alterations trigger dominant NF-κB activation and PI3K stimulation that are typical of the activated B cell (ABC) subtype of diffuse large B cell lymphoma (DLBCL) as well as mantle cell lymphoma (MCL) and chronic lymphocytic leukemia (CLL) (23). A second type of malignant signaling known as tonic BCR signaling, which is critical for healthy B cell survival (16), is chiefly driven by PI3K and the BCR (14, 15, 57). The mechanistic causes of this aberrant form of signal transduction are less well understood. Somatic mutations in BCR components are uncommon, but inactivation of the PI3K suppression factor phosphatase and tensin homolog (PTEN) is evident in many patients (24). Both aggressive Burkitt’s lymphomas (BL) and the GC B cell-like (GCB) DLBCL subtype have tonic signaling signatures (58, 59).

## Immunoregulatory co-receptors in B cell function and therapeutic targeting

Many activating and inhibitory surface receptors modulate B cell responses, but have also become targets for immunotherapy. For example, CD20 is a standard drug target in patients with a variety of B cell malignancies such as NHL, CLL, and BL (60, 61). The utility and safety of this approach in the clinic, used either as a single-agent monoclonal Ab (mAb) or in combination with TKIs and/or chemotherapy, has led to targeting other surface molecules including CD19, CD79B, CD22, and many others (62–64). However, the influence of transmembrane immunoregulators on the BCR and B cell functions is dynamic and reflects the relative cell surface organization of these proteins at rest and following antigen engagement (65–67). Over the past few years, the Reth laboratory has introduced new understanding of the nanoscale organization and localization of surface receptors. These relationships differ according to the membrane-bound Ig isotype (i.e., IgM vs IgD) at homeostasis versus activation (68, 69). For example, in resting cells CD19 and CD20 are situated with IgD, but following antigenic stimulation, reposition near IgM (70). In contrast, CD22, a receptor that bears immunoreceptor tyrosine-based inhibitory motifs (ITIM), forms BCR-independent homo-oligomers via *cis* interactions of α2,6-linked sialic acids at homeostasis, but is recruited into the antigen-receptor complex following activation (71). Thus, higher-order membrane organization and clustering interactions between co-receptors and the BCR are important for coordinating and regulating B cell functions. These associations also markedly differ when co-receptors (e.g. CD20) are synthetically deleted or targeted clinically (70), but are likely also altered when tumors downregulate target antigens to promote escape and therapeutic resistance (72). Thus, beyond their secondary structure and cytoplasmic features, the complex roles of immunoregulatory proteins on B cell functions also extend to their dynamic organization and integration with the BCR.

## Fc receptor-like family members

**Figure 1:**
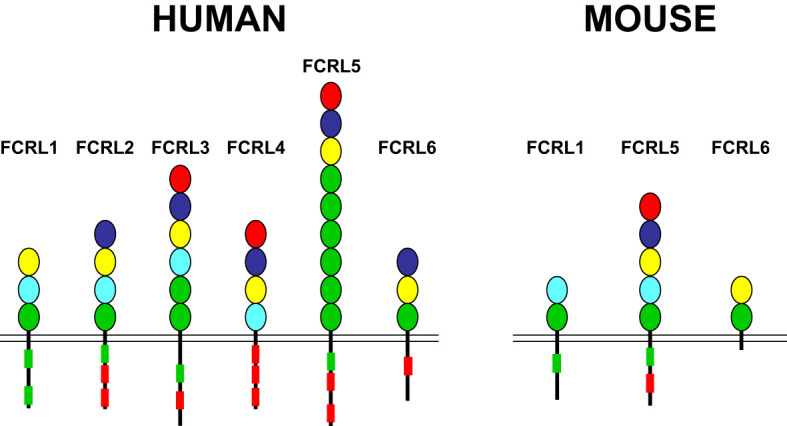
FCRL family members in humans and mice. FCRL1-6 type I transmembrane protein representatives in each species are shown with color-coded Ig-like extracellular domains that were defined according to their phylogenetic relationships (73). Cytoplasmic tails possess potential consensus ITIM (L/V/I)-X-Y-X-X-(L/V/I) (red boxes) and ITAM-like (E/D)-X-X-Y-X-X-(L/I)-X6-8-Y-X-X-(L/I) (green boxes) sequences. Note, despite the possession of two cytoplasmic tyrosines, FCRL6 in mice does not have consensus ITIM or ITAM-like sequences.

*FCRL1-6* gene family members are preferentially expressed by B cells and encode type I transmembrane glycoproteins that possess variable numbers of extracellular Ig-like domains and tyrosine-based motifs in their cytoplasmic tails (reviewed in (30, 73, 74)(**Figure 1**). The presence of intracellular ITIMs among most human (h) and mouse (m) FCRL proteins, suggests they predominantly suppress B cell functions. Indeed, dissection of FCRL2-5 tyrosine-based signaling has demonstrated their ability to be tyrosine phosphorylated, recruit SH2-domain containing tyrosine (SHP-1, SHP-2) and inositol (SHIP-1) phosphatases, and largely repress BCR-mediated whole-cell tyrosine phosphorylation, calcium flux, and other downstream pathways (75–79). While FCRLs are related to the classical Fc receptors for IgG and IgE that are either activating or inhibitory (80), additional cytoplasmic tyrosines that form hemi-ITAM or ITAM-like sequences in the tails of many FCRLs, indicates their regulatory properties are more complex. In fact, some FCRL proteins positively or negatively influence B cell responses according to the innate or adaptive nature of the stimulus, the operative type of cytoplasmic motif (ITAM vs ITIM), or in a cell subset-specific manner (79, 81–83).

Ligands have not yet been identified for hFCRL1, hFCRL2, or any of the mouse FCRLs. However, non-classical Fc-independent interactions for three hFCRLs with Ig have been found. These include binding of hFCRL3 to secretory IgA (84), hFCRL4 to systemic IgA (85), and hFCRL5 to IgG (86, 87). Although hFCRL6 is not expressed by B cells, but on cytotoxic T and NK cells (88, 89), it has been identified as a ligand for human leukocyte antigen (HLA)-DR/major histocompatibility class II (MHCII) molecules (90). Clearly, further discovery-based investigation is required to define the biologic counterparts for many FCRLs as well as the physiologic impact of their known associations.

## FCRL1 representatives in humans and mice possess co-activation features

**Figure 2:**
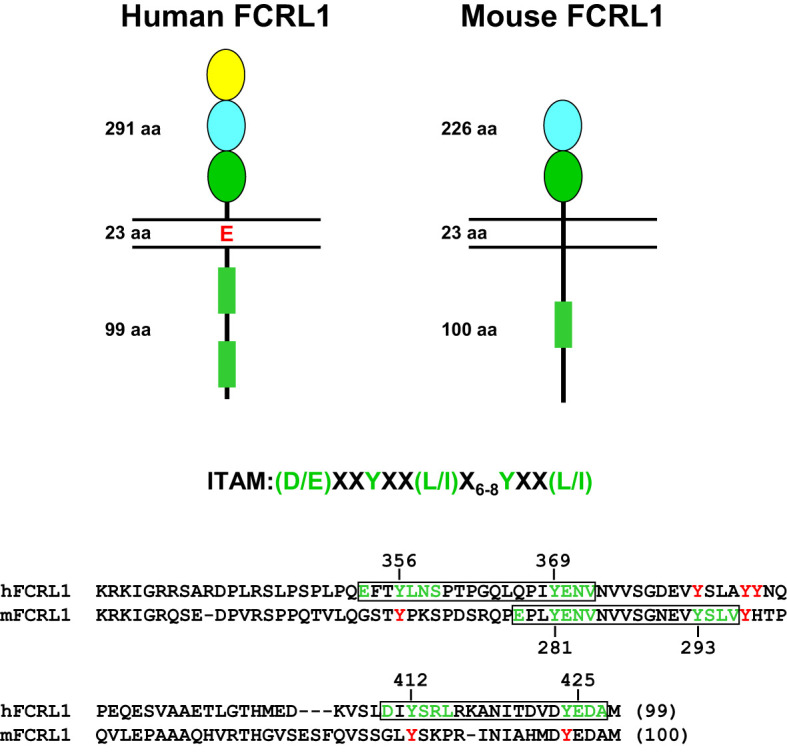
Structural features of human and mouse FCRL1. Extracellular, transmembrane and cytoplasmic amino acid (aa) lengths are indicated. Color-coding of extracellular Ig-like domain subtypes reflects relationships determined by phylogenetic analysis (73). Cytoplasmic sequences that resemble ITAM are detailed as rectangles (green). An ITAM consensus sequence is shown above an alignment of the human (hFCRL1) and mouse (mFCRL1) cytoplasmic tails. Implicated tyrosines and surrounding residues (green) that conform to ITAM-like sequences are numbered and boxed (black) as are other intracellular tyrosines (red) that do not fit a consensus. Note the mFCRL1 isoform 1 (shown) has an ectodomain 20 aa longer than isoform 2, which has an extracellular region of 206 aa.

Marked interspecies differences are found for the *FCRL* gene family in humans and mice (see **Figure 1**), which expanded and diversified since sharing a common ancestor more than 65 million years ago (91). Disparity is evident in gene number and representation as well as genetic organization and primary structure (73, 92). Like other multigene families encoding immune receptors, species-specific genetic variation may reflect evolutionary changes in their ligand(s) and/or adaption and subfunctionalization to pathogenic pressure that is advantageous to host immunity and survival (93–95). The conservation of *FCRL1* orthologs in both species seems to underscore this representative’s biological importance (**Figure 2**). In humans, *FCRL1* encodes a type I transmembrane glycoprotein with three extracellular Ig-like domains, a transmembrane region harboring a charged glutamic acid (E) residue, and a 99 amino acid (aa) cytoplasmic tail with seven tyrosines (96–98). Mouse FCRL1 possesses two Ig-like extracellular domains and a 100 aa cytoplasmic tail with six tyrosines. However, the amino-terminal Ig-like domain and charged transmembrane residue both present in its human counterpart are absent in mice (99). Despite similarity with the classical Fc receptors for IgG, hFCRL1 does not bind Ig, and ligand(s) for it remain unknown (73, 100). In contrast to FCRL2-5, which all have one or more cytoplasmic consensus ITIM sequences, human and mouse FCRL1 lack this characteristic. Instead, they share 43% intracellular aa identity and have ITAM-like sequences. Orthodox ITAMs in CD79A/B and CD3ζ have an amino-terminal acidic residue followed by two repeats of the consensus sequence Y-X-X-L/I separated by 6–8 amino acids (E/D)-X-X-Y-X-X-(L/I)-X_6–8_-Y-X-X-(L/I) (101). Similar motifs are present in human and mouse FCRL1 (30), but with some differences. These mainly relate to the aliphatic residues at the +3 position relative to the tyrosines (Y). In the case of mFCRL1, which has a single ITAM-like sequence, there are valines (V) rather than leucine (L) or isoleucine (I) at these sites (99). However, hFCRL1 has two ITAM-like sequences (96). These also vary at the +3 residue, with a polar serine (S) and V in the first motif and an alanine (A) at the last position of the second ITAM-like sequence. These features suggest these FCRL1 proteins possess activating function, but likely vary in the types of cytoplasmic elements they recruit compared to canonical ITAM found in B or T cell antigen-receptor signaling subunits.

## FCRL1 is differentially expressed during B cell development and promotes humoral responses

Transcripts for *FCRL1* in humans and mice are present in the bone marrow and secondary lymphoid tissues (96, 97, 99). The generation of hFCRL1-specific mAbs by several groups (100, 102, 103), enabled staining of the receptor by flow cytometry. During B lymphopoiesis, hFCRL1 is first detected at low levels on precursor B cells in the bone marrow, but its surface density increases as cells express IgM heavy chains. In the circulation, hFCRL1 is a pan-B cell marker restricted to CD19^+^ B cells, but is absent from monocytes, T, and NK cells (100). Staining of human tonsils reveals peak expression on naïve B cells (IgD^+^CD38^-^), but hFCRL1 declines as the BCR is downregulated from the surface during Ig diversification and affinity maturation on antigen-activated pre-GC (IgD^+^CD38^+^) and GC (IgD^-^CD38^+^) cells. As B cells terminally differentiate, FCRL1 reemerges on memory cells (IgD^-^CD38^-^), albeit at slightly lower levels than naïve B cells. In contrast, plasma cells (IgD^-^CD38^hi^) express little hFCRL1 (100). Preferential naive B cell expression is also consistent with *in situ* hybridization analysis of human tonsillar tissue that shows mantle zone enrichment of *FCRL1* transcripts (97). Recent single cell RNA-seq studies validate this general expression pattern, but also indicate that *FCRL1* transcripts are tightly regulated during distinct stages of GC B cell development, including dark and light zone centroblast and centrocyte B cell subsets that are implicated in the generation of lymphomas and leukemias (104). In mice, transcript analyses and recent work at the protein level show a similar distribution for mFCRL1 [(99, 105) and unpublished studies]. The mouse counterpart is also initially detected on bone marrow precursor B cells, but increases as a function of maturation with recirculating cells displaying higher mFCRL1 surface levels. Among splenocytes, mFCRL1 is a pan-B cell marker that is expressed by all transitional B cell stages and follicular and marginal zone B cells, but is downregulated by GC B cells and plasma cells (105). Thus, the developmental regulation of FCRL1 during B cell differentiation appears to be conserved interspecies.

Two *Fcrl1* deficient mouse models have recently been reported. In one, Zhao et al. used a clustered regularly interspaced short palindromic repeats (CRISPR)-editing approach to target the fourth and tenth exons of *Fcrl1* in the C57BL/6 strain that encode the second Ig-like extracellular domain and the amino terminus of the cytoplasmic tail (106). In another, DeLuca et al. examined a homologous recombination model in 129S1/SvImJ background mice that deleted exons 2-6, which encode the second portion of the split signal peptide through the transmembrane region (105). Both studies failed to find developmental differences for B cells from the precursor through mature stages in the bone marrow. However, alterations were evident among peripheral B cell subpopulations in the spleen. Though subset frequencies at homeostasis appeared similar between these studies, DeLuca et al. found slightly higher numbers of immature and mature B cells in the absence of mFCRL1 (105). This could be attributed to significantly increased numbers of transitional stage III (T3), follicular, and marginal zone B cells. While this phenotype could reflect differences in the targeting strategy and/or strain background, it suggests mFCRL1 may modulate stage-dependent peripheral B cell accumulation in the spleen. Unlike CD19, another broadly expressed surface antigen that is critical for peripheral B cell development (107), these findings show that B cell maturation is largely intact in both *Fcrl1* mutant models.

These studies reveal that FCRL1 may preferentially influence later steps of B cell maturation during Ig diversification and terminal differentiation that are driven by adaptive responses in the GC microenvironment. This possibility is supported by experiments showing that GC B cell frequencies in *Fcrl1*^-/-^ mice were significantly lower one week after immunization with sheep red blood cells (106). Humoral responses to hapten-conjugated (4-hydroxy-3-nitrophenylacetyl, NP) T cell-independent type II (TI-2) and T cell-dependent (TD) antigens also showed defects. NP-specific IgM and IgG3 titers against TI-2 Ficoll were both lower in *Fcrl1*^-/-^ deficient mice one week after challenge (106). Primary TD antigen-specific IgM and IgG (high and low affinity) responses to immunization with NP-KLH in alum were also significantly reduced at 7 and 14 days. Moreover, numbers of NP-specific IgM and IgG plasmablasts were correspondingly lower in *Fcrl1*^-/-^ mice 3 and 6 days following immunization. Importantly, DeLuca et al. corroborated similar defects in antigen-specific humoral responses in their *Fcrl1*^-/-^ mouse studies (105). Thus, consistent with its activating features, these findings indicate that mFCRL1 promotes TI and TD antigen-specific B cell responses. However, secondary responses still require investigation in these models. **Table 1** summarizes emerging knowledge of FCRL1 functions in humans and mice.

**Table 1:** Studies investigating FCRL1 function in human and mouse models.

Model	Perturbation/ Stimulus	Predicted role/function	Reference
Human			
Tonsillar B cells	α-FCRL1 mAb/ α-IgM pAb	- Promotes activation (CD69, CD86) and proliferation upon engagement - Enhances BCR-induced proliferation	Leu (100)
Daudi BL B cell line	α-FCRL1 mAb/ α-IgM pAb	- Co-ligation augments BCR calcium signaling	
A20IIA1.6 B cell line	α-FCRL1 mAb	- FCRL1 tyrosine phosphorylation	
Ramos & Daudi BL B cell lines; DLBCL samples	shRNA depletion	- Promotes proliferation and survival - Potentiates PI3K/AKT and p65 NF-κB signaling	Yousefi (108, 109)
Mouse			
*Fcrl1*^-/-^ (C57BL/6) Splenic B cells	SRBC NP-Ficoll (alum) NP-KLH (alum) α-IgM pAb	- Promotes GC B cell formation (day 7) - Promotes T cell–independent type II Ab responses (day 7) - Promotes T cell–dependent Ab responses (day 7 and 14) - Drives NP-specific IgM and IgG extrafollicular plasmablast formation (day 3 and 6) - Potentiates BCR and calcium signaling, and proliferation	Zhao (106)
*Fcrl1*^-/-^ CH-27 B cell line	PC α-HA mAb α-IgM pAb	- Potentiates calcium signaling - pY281 dependent c-Abl recruitment - Promotes synaptic recruitment of pSyk, pBLNK, and pPI3K (p85α)	
*Fcrl1*^-/-^ (129S1/SvImJ) Splenic B cells	Homeostasis Not specified Pervanadate α-Ig pAb	- Limits splenic T3, follicular, and marginal zone B cell numbers - Promotes T cell-dependent and independent Ab responses - pY281 dependent GRB2 recruitment - Inhibits ERK phosphorylation - Potentiates BCR-mediated calcium signaling	DeLuca (105)
*Fcrl1*^-/-^ A20IIA1.6 B cell line	Pervanadate α-κ mAb beads α-Ig pAb	- pY281 dependent GRB2 recruitment - Inhibits ERK phosphorylation - Potentiates BCR-mediated calcium signaling	

α, anti; Ab, antibody; mAb, monoclonal Ab; pAb, polyclonal Ab; BCR, B cell receptor; BL, Burkitts lymphoma; shRNA, short hairpin RNA; PI3K, phosphoinositide 3-kinase; AKT, serine/threonine-protein kinase B; NF-κB, Nuclear factor kappa-light-chain-enhancer of activated B cells; SRBC, sheep red blood cells; GC, germinal center; NP, 4-hydroxy-3-nitro-phenylacetyl; KLH, keyhole limpet hemocyanin; Ig, immunoglobulin; PC, phosphorylcholine; HA, hemagglutinin; c-Abl, Abelson non-receptor tyrosine kinase; pSyk, phospho-spleen-associated tyrosine kinase; pBLNK, phospho-B cell linker protein; T3, transitional stage 3; GRB2, growth factor receptor-bound protein 2; ERK, extracellular signal-regulated kinase.

## FCRL1 promotes B cell activation and proliferation

Several groups have examined FCRL1 regulatory properties in B cells. Early work used receptor-specific mouse anti-human FCRL1 mAbs as a surrogate ligand for stimulating purified tonsillar B cells *in vitro* (100). Ligation of biotinylated-Fab fragments with streptavidin significantly induced CD69 and CD86 surface expression and downregulated IgD expression after 48 hours. In proliferation assays, hFCRL1 engagement alone stimulated ^3^H-thymidine uptake in a concentration-dependent manner, but also enhanced BCR-mediated proliferation when co-ligated with suboptimal concentrations of anti-IgM Abs. Thus, consistent with its ITAM-like characteristics, hFCRL1 can exert positive effects in B cells that are independent as well as co-incident with the BCR.

Using a retroviral short hairpin RNA (shRNA)-targeting strategy to deplete *FCRL1* in the Ramos and Daudi BL cell lines (96, 100), Yousefi et al. showed decreased cell proliferation by carboxyfluorescein diacetate succinimidyl ester (CFSE) labeling as well as increased apoptotic cell death over 48-96 hours (108). These studies did not assess the impact of BCR stimulation, but 48 hours after *FCRL1* knockdown, transcripts encoding the anti-apoptotic gene B cell lymphoma 2 (*BCL2)* were lower, whereas the pro-apoptotic BH3 interacting-domain death agonist (*BID)* and BCL2-associated X protein (*BAX)* genes rose. A follow up report using this shRNA approach in primary DLBCL patient samples indicated similar defects in proliferation and apoptosis (109). Cell cycle analysis by propidium iodide staining showed that frequencies of G2/M cells were reduced, but sub-G1 and G1 cells were increased in hFCRL1-depleted cells after 72 hours, indicating higher levels of apoptosis. Although the rigor of these *FCRL1* deficiency studies (108, 109), with respect to targeting efficiency and numbers of replicate experiments are somewhat unclear, the results are consistent with observations of positive roles for FCRL1 in B cell proliferation and survival.

In mice, BCR ligation in *Fcrl1* deficient primary splenic B cells also demonstrated impaired proliferation as determined by lower CSFE dilution compared to wild-type (WT) control B cells (106). However, these *ex vivo* findings differ from those observed *in vivo* by DeLuca et al. (105), who found relatively increased numbers of splenic T3, follicular, and marginal zone subsets in the absence of mFCRL1. This phenotype might alternatively suggest that mFCRL1 limits proliferation or biases differentiation. While incorporation of the thymidine analog 5-bromo-2’-deoxyuridine (BrdU) was significantly higher in *Fcrl1^-/-^* splenic transitional, marginal zone, and GC B cells, DNA content by cell cycle analysis did not show differences in the frequencies of cells in the S/G2/M phases (105). These data seem to refute findings that mFCRL1 deficiency enhances B cell proliferation, but apoptosis studies indicated the frequencies of immature and mature follicular B cells undergoing cell death were lower compared to WT mice. Data from this particular model, indicating differential roles for mFCRL1 in proliferation and survival, could imply variable properties for this receptor in selection and peripheral tolerance during homeostasis versus host defense. However, based on the current limited studies, much more work is required to define physiologic functions for FCRL1 interspecies and in various immune contexts.

## FCRL1 enhances B cell signaling and formation of the BCR-signalosome

**Figure 3:**
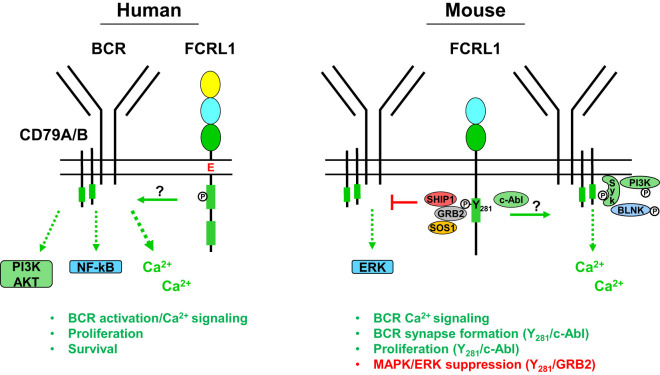
Potential signaling relationships for FCRL1 in humans and mice with the B cell receptor (BCR). A schematic summary of proximal and downstream signaling features based on current understanding of FCRL1 effector functions in human and mouse B cells. Roles for hFCRL1 in regulating B cell signaling cascades have been investigated by knockdown studies in human cell lines revealing lower NF-κB (p65) and AKT (S473) activation, reduced proliferation, and increased apoptosis. Positive effects were found by ligating hFCRL1 with receptor-specific antibodies, which induce its tyrosine phosphorylation as well as promote tonsillar B cell proliferation and activation marker expression. Moreover, hFCRL1 co-ligation with the BCR augments B cell proliferation and calcium flux. Currently, proximal effector-recruitment relationships remain largely undefined for hFCRL1. Emerging understanding of modulatory properties for mFCRL1 appear more complex. Two independent studies using primary B cells from *Fcrl1*^-/-^ mice along with deficient cell lines demonstrate that mFCRL1 potentiates BCR-mediated calcium signaling as well as T cell-independent and dependent humoral responses. However, findings differ with respect to the elements recruited to the cytoplasmic Y281 residue and its functional impact. Zhao et al. (106) have identified an Y281 phosphorylation-dependent interaction with c-Abl that promotes synaptic recruitment of the BCR with pSyk, pBLNK, and pPI3K (p85α) and stimulates B cell proliferation. However, DeLuca et al. (105) found that Y281 can directly bind the GRB2 adapter protein, which in turn recruits the SOS1 guanine nucleotide exchange factor and SHIP1 phosphatase resulting in MAPK/ERK suppression. These findings portend complex roles for FCRL1 as a proximal regulator that has variable influence on B cell signaling responses in different contexts.

Signaling studies to examine roles for FCRL1 and its ITAM-like properties as a proximal regulator in B cells have been performed in humans and mice (**Figure 3**). Early work investigated the influence of hFCRL1 on calcium signaling in FCRL1^+^ Daudi BL cells (100). Though mAb-mediated ligation of hFCRL1 alone did not impact calcium flux, co-ligation with IgM showed enhanced effects on intracellular calcium mobilization over BCR stimulation alone. There is also evidence for hFCRL1 having positive effects on other downstream pathways. Following *FCRL1* shRNA knockdown in BL cell lines and primary DLBCL cells, Yousefi et al. found downregulation of both phosphorylated (p)-AKT (S473) and p-p65-NF-κB by intracellular staining and flow cytometry analysis (108, 109). While these studies require biochemical confirmation, they implicate hFCRL1 in promoting signaling pathways important for B cell survival and effector function.

Recent work has more extensively probed FCRL1 signaling in mice. Stimulatory effects for mFCRL1 on BCR-mediated calcium signaling have been shown in CRISPR-edited cells. Using *Fcrl1* deficient C57BL/6 primary B cells or CH-27 cells, an innate-like B-1 B cell line expressing a BCR that binds phosphorylcholine (PC), Zhao et al. found that compared to WT cells, calcium signaling was reduced when the BCR was triggered with either polyclonal anti-IgM Abs or the cognate CH-27 PC antigen (106). BCR surface levels were not altered by the loss of mFCRL1 in either B cell type. Similar observations were made by DeLuca et al. who also found reduced calcium mobilization following BCR stimulation in *Fcrl1* deficient 129S1/SvImJ primary B cells or the class-switched IgG2a mouse memory B cell line A20IIA1.6 (105). These findings indicate that the expression of mFCRL1 itself potentiates BCR signaling.

To investigate the proximal impact of mFCRL1 on the formation and recruitment of signaling elements to the BCR synapse, Zhao et al. used total internal reflection fluorescence microscopy (TIRFM) imaging (106). Microscopy was performed with CRISPR-edited CH-27 and primary B cells from C57BL/6 mice to quantitate biophysical effects of mFCRL1 deficiency on BCR activation (106). In these experiments, the BCR was stimulated with F(ab’)_2_ fragments embedded on the surface of lipid bilayers for 10 minutes. Calculations of mean fluorescence intensities by TIRFM disclosed significantly impaired synaptic accumulation of the BCR as well as pSyk, pBLNK, and pPI3K (p85α) in CH-27 and primary B cells lacking mFCRL1 (106). However, these defects could be rescued by transduction with WT mFCRL1. Using amino-terminal hemagglutinin (HA) tagged and carboxyl-terminal fluorescent labeled chimeric proteins, the distribution of mFCRL1 relative to the BCR was also traced in CH-27 and primary splenic B cells. By TIRFM imaging, anti-HA ligation induced aggregation of mFCRL1 but not the BCR, whereas BCR ligation alone or together with HA strongly promoted the synaptic accumulation of both receptors (106). Thus, BCR engagement induces passive mFCRL1 recruitment, accumulation, and co-localization with the BCR in the immunologic synapse. While mFCRL1 ligation alone promotes self-aggregation, it does not appear to promote synapse formation. Hence, mFCRL1 independently potentiates and promotes activation and synapse formation directed by BCR engagement. Given interspecies transmembrane and cytoplasmic differences, future studies will need to explore how conserved the relationships are for FCRL1 in BCR synapse formation.

## Contributions of cytoplasmic tyrosine-based motifs to FCRL1 B cell regulation

Evidence that hFCRL1 can be tyrosine phosphorylated was first shown by transducing the A20IIA1.6 B cell line with a HA-tagged variant. HA immunoprecipitation and Western blotting analyses of these cells, stained with anti-FCRL1 biotinylated-Fab mAb fragments and cross-linked with streptavidin, showed the induction of tyrosine phosphorylation at 2 minutes and 15 minutes, albeit at lower levels at the later time point (100). While more detailed effector-recruitment and downstream analyses await hFCRL1, greater progress has been made on the intracellular properties of FCRL1 in mice.

As detailed in **Figure 2**, mFCRL1 has six cytoplasmic tyrosines including a relatively canonical ITAM consensus sequence. Notably, there are four *Fcrl1* splice isoforms in mice. Isoform 1 is the longest at 363 aa. Among the three shorter variants, isoform 3 (300 aa) lacks a transmembrane region and could be secreted (99). Accordingly, the numbering of cytoplasmic tyrosine residues differs by variant. The ITAM tyrosines in isoform 1 are at aa positions 301 and 313. However, two signaling studies discussed below, apply the numbering of ITAM tyrosines at residues 281 and 293 found in isoforms 2 (343 aa) and 4 (324 aa).

To identify potential docking sites for SH2 domain-containing signaling proteins in the mFCRL1 cytoplasmic tail, Zhao et al. used a database screen to identify the Abelson tyrosine kinase c-Abl as a candidate for targeting the Y_281_ENV motif (106) (see **Figure 2**). To assess this, TIRFM imaging was performed on *Fcrl1*^-/-^ CH-27 B cells doubly-transduced with cDNAs encoding fluorescently-labeled HA-tagged WT FCRL1 and c-Abl chimeric proteins. Anti-HA ligation induced co-localization of the proteins in microclusters, but a Y281F mutation impaired this association, as well as synaptic recruitment of the BCR, pSyk, pBLNK, and pPI3K (p85α). Biochemical analysis showed that BCR or HA-tag ligation induced tyrosine phosphorylation of WT mFCRL1 in CH-27 B cells, but this effect was markedly impaired for the Y281F mutant. However, the detection of low residual tyrosine phosphorylation in Y281F precipitates implicated signaling roles for other tyrosines in the mFCRL1 tail. In pull-down studies of GST-tagged c-Abl in BCR or anti-HA ligated CH-27 cells, mFCRL1 binding to c-Abl was lost with the Y281F mutant. An endogenous association for c-Abl with mFCRL1 in CH-27 was also Y281-dependent. ELISA-based binding assays using synthetic peptides of cytoplasmic mFCRL1 aa residues 266-296, including the ITAM (Y_281_ENV and Y_293_SLV), showed the c-Abl interaction with Y281 was indeed phosphorylation-dependent. Finally, functionality of the mFCRL1 Y281 residue was also suggested by cell proliferation studies with primary B cells, showing impaired CSFE dilution following anti-HA ligation in Y281F mutants versus WT transductants or WT primary cells treated with the c-Abl TKI imatinib.

These studies provide several lines of evidence that Y281 c-Abl recruitment provides mFCRL1 with activation properties. However, DeLuca et al. were unable to independently replicate these results in the A20IIA1.6 B cell line that endogenously expresses c-Abl (105). While other factors could differ between the model systems employed by these groups, it is possible that overexpression of transduced proteins could augment binding promiscuity, the c-Abl/mFCRL1 interaction is of low affinity, or is context dependent. This latter possibility could extend to differences in BCR isotype. For example, primary B cells and CH-27 cells express surface IgM, whereas A20IIA1.6 B cells are class-switched for IgG. Thus, further in-depth biochemical studies are needed to investigate this disparity for the c-Abl/mFCRL1 interaction as well as associations with other elements that equip mFCRL1 with regulatory function.

In a second set of studies to dissect the tyrosine-based signaling properties of mFCRL1, DeLuca et al. used an agnostic biochemical approach (105). Lysates from pervanadate-treated A20IIA1.6 B cell line transductants, expressing either FLAG-tagged WT mFCRL1 or a tail variant mutating all six tyrosines (Y6F), were subjected to immunoprecipitation and liquid chromatography coupled to tandem mass spectrometry. This strategy identified phosphorylation of the Y281 and Y297 residues along with potential associations for the adaptor proteins GRB2 and GRAP, the inositol phosphatase SHIP-1, and the guanine nucleotide exchange factor SOS1. To interrogate these relationships, CRISPR-edited *Fcrl1*^-/-^ A20IIA1.6 B cells were generated and transduced with mFCRL1 WT, Y281F, Y297F, and Y6F mutants. Interestingly, despite an expected MW of ~36 kDa, Western blotting of pervanadate-treated immunoprecipitates for tyrosine phosphorylation status, FLAG, or FCRL1 itself, demonstrated that even under reducing conditions mFCRL1 migrated at a MW of ~72 kDa (105). This suggested that the protein may exist as a stable SDS-resistant dimer and/or undergoes post-translational modification. Accordingly, the two Ig-domains of FCRL1 in mice have five potential N-linked glycosylation sites, but these studies did not assess the impact of glycosidase treatment.

While the mFCRL1 Y281 residue appeared critical for tyrosine phosphorylation following pervanadate treatment and the associations of GRB2, SHIP-1, and SOS1 (see **Figure 3**), recruitment of these effectors differed upon BCR stimulation. After BCR triggering, the GRB2 association remained intact, but SHIP-1 only weakly bound, and SOS1 was no longer detected. Thus, elements recruited to the mFCRL1 tail may vary according to the type and strength of stimulus. Unfortunately, an association for GRAP could not be confirmed due to a lack of available reagents. Because GRB2 and SHIP-1 can directly associate (110), the investigators generated CRISPR-edited *Grb2*^-/-^ A20IIA1.6 B cells and confirmed a direct interaction between mFCRL1 and GRB2. These findings indicate that GRB2 may serve as a docking site for SHIP-1 and SOS1 enabling indirect associations with the mFCRL1 scaffold.

Because GRB2 and GRAP can potentiate MAPK/ERK phosphorylation in human B cells (111), DeLuca et al. analyzed intracellular pERK activation by flow cytometry in WT A20IIA1.6 cells and a panel of *Fcrl1*^-/-^ transductants following BCR stimulation with anti-κ light chain beads. Surprisingly, pERK levels were highest in *Fcrl1*^-/-^ deleted cells at 10 minutes, but lowest in transductants rescued with WT mFCRL1. Suboptimal pERK inhibition was found for the Y281F and Y297F mutants, indicating that both residues may contribute to the repression of this pathway. To confirm the role of GRB2, similar studies were performed with *Grb2*^-/-^ and *Fcrl1*^-/-^ edited cells that showed comparable intracellular pERK levels. Moreover, introduction of an mFCRL1-GRB2 fusion construct restored pERK inhibition in *Grb2*^-/-^ A20IIA1.6 B cells. Studies with primary splenocytes from unchallenged WT and *Fcrl1*^-/-^ mice validated suppressive effects for mFCRL1 on pERK following F(ab’)_2_ BCR engagement *ex vivo*. Immature and mature B cells from *Fcrl1*^-/-^ mice both showed significantly higher levels of pERK compared to WT counterparts. Surprisingly, analysis of pSyk, pPI3K (p85/p55), and pAKT (S473) did not show significant differences following BCR stimulation in A20IIA1.6 transductants or primary B cells. However, trends of lower pSyk and pPI3K were evident in *Fcrl1*^-/-^ primary B cells. These findings indicate that mFCRL1 suppresses BCR-mediated pERK production. However, systemic defects in antigen-specific humoral responses found for two independent *Fcrl1*^-/-^ models, as well as proliferation and calcium signaling, strongly indicate mFCRL1 also serves positive roles in B cells. Perhaps like other family members with complex dual properties (79, 81–83), mFCRL1 exerts differential regulation that varies according to the type of stimulus, developmental stage, and microenvironmental context. Further study in human and mouse models will be required to investigate these possibilities.

## hFCRL1 is broadly expressed in B cell lymphoproliferative disorders

Its restricted distribution among B lineage cells, tightly regulated expression by GC subsets, complex tyrosine-based signaling properties, and promotion of cellular and systemic humoral responses, collectively implicate a role for hFCRL1 in transformed B cells. Initial studies detected *FCRL1* expression in human leukemia and NHL cell lines, including BLs (96, 97). A query of the ‘Lymphochip’ and related microarray work by the Staudt group demonstrated differential overexpression of *FCRL1* transcripts by primary follicular lymphoma (FL), CLL, DLBCL, and MCL samples (22, 112). These data indicated that *FCRL1* is broadly expressed among indolent and aggressive B cell lymphoproliferative disorders. Subsequent studies have identified *FCRL1* transcripts in other mature B cell malignancy samples (113, 114). However, consistent with its distribution pattern during normal B cell development, *FCRL1* expression appears to be lower in cases of acute lymphoblastic leukemia (ALL), which derives from bone marrow B cell precursors (115), and multiple myeloma that originates from transformed plasma cells (116).

Using receptor-specific mAbs for flow cytometry analysis, studies have confirmed hFCRL1 protein expression on the surface of different B cell malignancies. The detection of hFCRL1 on CLL samples was reported by several groups and was present at higher relative levels than FCRL2-5 (102, 117). Our laboratory generated a panel of hFCRL1-5 mAbs to analyze 107 cryopreserved CLL samples for comparisons with known prognostic markers including *IGHV* mutation status (118), CD38 and ZAP-70 expression (119), and clinical parameters (120). Except for hFCRL4, all other family members were found on CLL cells and hFCRL1 had the highest relative density (121). This work also found high concordance for hFCRL2 expression with favorable *IGHV* (mutated CLL) (121) and cytogenetic status (122). Du et al. found hFCRL1 on a series of GCB-DLBCL and BL cell lines as well as many primary FL, MCL, hairy cell leukemia, and BL patient samples (117). Collectively these findings endorse hFCRL1 as an intriguing molecule for further mechanistic and therapeutic studies in B cell lymphoproliferative disorders.

## Potential prognostic value of hFCRL1 in aggressive non-Hodgkin’s lymphomas

**Figure 4:**
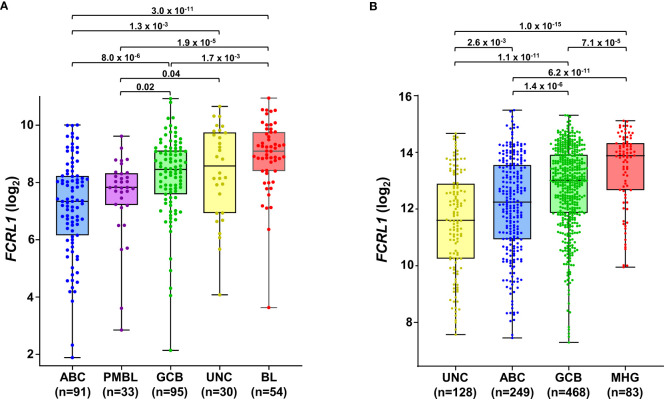
*FCRL1* is upregulated by mature non-Hodgkin’s B cell lymphomas (NHL) and correlates with more aggressive disease. Box-and whisker plots of log_2_ transformed *FCRL1* transcript expression in NHLs including: activated B cell-like (ABC) diffuse large B cell lymphoma (DLBCL), primary mediastinal B cell lymphomas (PMBL), germinal center-B cell like (GCB) DLBCL, unclassified lymphomas (UNC), molecular high-grade (MHG) lymphomas, and Burkitt’s lymphomas (BL) classified according to gene expression profiling and analyzed using the R2 microarray platform (http://r2.amc.nl). Data are derived from **(A)** GSE4732 (n=303) (125) and **(B)** GSE117556 (n= 928) (126). Sample numbers are indicated in parentheses and *P* values were calculated by ANOVA with Welch’s correction.

Even though it is overexpressed by many different leukemias and lymphomas, there is little evidence for *FCRL1* as a high-frequency target of recurrent mutation or genetic alteration in the catalogue of somatic mutations in cancer (COSMIC) database or other genomic analyses of lymphomas (25, 27, 123, 124). However, given its activating features and expression by DLBCL and BL NHLs, it is possible that hFCRL1 might be a useful biomarker to provide prognostic or pathologic insight into these malignancies. To examine this, we analyzed *FCRL1* expression in 303 NHL samples from Dave et al. who performed sub-classification by gene expression profiling (GEP) (125). **Figure 4A** shows that *FCRL1* is most significantly upregulated by BL followed by unclassified lymphomas, GCB-DLBCL, and primary mediastinal B cell lymphomas, with ABC-DLBCL having relatively lower expression. These findings provide evidence that higher *FCRL1* transcript levels correlate with aggressive lymphomas characterized by pathologic tonic signaling (19). For validation, we analyzed *FCRL1* expression by GCB- versus ABC-DLBCL subtypes from three other GEP studies (126–128). These data consistently showed that mean *FCRL1* expression (log_2_) by GCB-DLBCL subtype tumors was more than one-fold higher than transcript levels found in ABC-DLBCL tumors (**Table 2**).

**Table 2:** Relative *FCRL1* expression by GCB- and ABC-DLBCL subtypes.

Study	GEO Accession	GCB-DLBCL (n)	ABC-DLBCL (n)	Total (n)	GCB : ABC (mean log_2_)	*P* value
Dave (125)	GSE4732	95	91	186	1.14	8 x 10^-6^
Xiao (127)	GSE10846	183	167	350	1.13	2.7 x 10^-5^
Roche (128)	GSE31312	237	214	451	1.08	5.4 x 10^-5^
Sha (126)	GSE117556	468	249	717	1.05	1.4 x 10^-6^

Data analyses were performed and plots generated using the R2 microarray platform (http://r2.amc.nl).

P values were calculated by ANOVA with Welch’s correction.

GCB, germinal center B cell-like; ABC, activated B cell; DLBCL, diffuse large B cell lymphoma.

**Figure 5:**
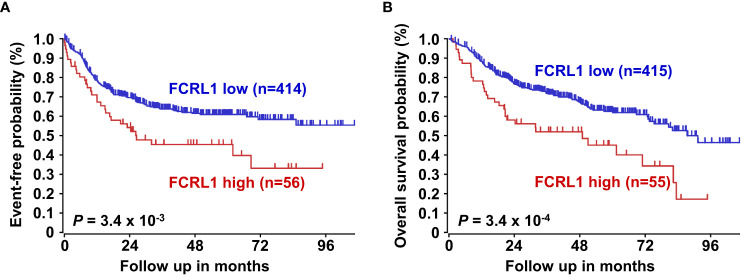
Higher *FCRL1* expression predicts worse event-free and overall survival in diffuse large B cell lymphoma (DLBCL). Kaplan-Meier plots of event-free **(A)** and overall survival **(B)** for DLBCL patients (n=470) segregated by *FCRL1* transcript levels (GSE31312) (40). Data analyses and plots were generated using the R2 microarray platform (http://r2.amc.nl). *P* values were determined by the log-rank test.

Even with dramatic progress in determining molecular factors responsible for the pathogenesis of DLBCL, biologic heterogeneity remains (22, 25–29). In fact, high-grade lymphomas with intermediate BL-like phenotypic features have been described with poor-risk features that would benefit from improved classification and precision therapies. A recent analysis of 928 patients by Sha et al. defined a molecular high-grade (MHG) group by GEP that largely overlapped with the GCB-DLBCL subtype, but exhibited more proliferative features (126). In this study, progression-free survival in MHG patients treated with chemoimmunotherapy (R-CHOP regimen) was nearly 2-fold lower at 3 years compared to other disease types. Patients with double-hit myelocytomatosis bHLH transcription factor *(MYC)* and *BCL2* and/or B-cell lymphoma 6 (*BCL6)* gene rearrangements were included among MHG samples, but represented only half of the total. Interestingly, a query of samples from this study revealed significantly higher *FCRL1* transcripts in MHG samples followed by GCB-DLBCL, ABC-DLBCL, and unclassified lymphoma samples (126) (**Figure 4B**). Because these data implicate *FCRL1* upregulation with more aggressive disease, we also assessed whether it correlates with clinical outcomes in DLBCL. In a cohort of 498 DLBCL patients for which clinical and GEP data were available (128), we found that higher *FCRL1* expression predicted significantly worse disease progression and survival (**Figure 5**). Although confirmation at the protein level in well-annotated primary samples is required, these data merit further dissection of FCRL1 as biomarker in the pathogenesis and treatment of aggressive lymphomas.

## Exploring hFCRL1 as a novel immunotherapeutic candidate

Despite its wide expression by B cell malignancies and logical potential as an immunotherapeutic target, few preclinical studies have explored this aspect of hFCRL1. Rather the hFCRL5 family member has become a promising target for Ab-mediated therapy. Currently, an hFCRL5-CD3 bispecific T cell engager (BITE) is in clinical trials (NCT03275103) for patients with relapsed or refractory multiple myeloma (129–131). However, hFCRL5 showed limited utility when targeted with a single-agent mAb toxin conjugate in myeloma (132). Early preclinical studies by Du et al. explored targeting hFCRL1 with a 38-kDa fragment of Pseudomonas exotoxin A (PE38) fused to single-chain variable fragments (scFv) cloned from mAbs to produce recombinant immunotoxins (117). This strategy has been successful for several antigens including CD22 in hairy cell leukemia (133). Two anti-hFCRL1 specific immunotoxins, E3(Fv)-PE38 and E9(Fv)-PE38, showed selective activity against hFCRL1-expressing DLBCL and BL cell lines (117). Binding affinities were in the nanomolar range, stable over time, and cytotoxicity correlated with hFCRL1 expression levels. The investigators note that these characteristics were comparable to the cytotoxic activity of other immunotoxins examined in clinical trials and therefore provide support for pursuing hFCRL1 as an immunotherapeutic target.

A potential role for hFCRL1 in this arena seems more likely especially because other commonly targeted B cell-restricted antigens may become modulated or lost from the cell surface in patients over time. For example, downregulation leading to immunotherapeutic resistance in patients is an escape mechanism that has been observed following anti-CD20 mAb treatment as well as chimeric antigen receptor (CAR) T cell-based therapies directed at CD19 or CD22 (72, 134–137). The application of multi-specific CAR-T and mAb strategies, where more than one surface receptor is concurrently targeted in *cis* on the same cells or in *trans* via intercellular engagement, such as BITEs being exploited for hFCRL5, may provide alternatives that lower the risk of immune-evasion mechanisms by malignancies (21, 136, 138–143). Thus, a number of therapeutic avenues remain open for the hFCRL1 antigen in malignant B cell disorders particularly as its functional and mechanistic properties become better understood.

## Concluding remarks

Apart from the interspecies diversity of FCRL1 representatives in humans and mice, their conserved B cell distribution and tyrosine-based activating characteristics suggest that parallel investigation will be helpful for unraveling their complex functions. Physiologic roles for FCRL1 in regulating humoral immunity are indicated by similar stage-specific modulation on B cell subsets during GC-based diversification in lymphoid tissues as well as impaired responses to antigenic challenge in mutant mouse models. However, FCRL1 regulatory properties also appear to differ at homeostasis versus activation. Given the seeming intricacies of FCRL1 and the current limited findings in this nascent field, it may not be surprising that some conflicting results reviewed here will require further experimental validation to reach clarity. Identification of the FCRL1 ligand(s) should be very informative for understanding its complicated biology. Beyond host defense, broad expression by B cell malignancies and NHLs, which largely derive from transformed GC B cells, implicate hFCRL1 as potential pathogenic factor. Although it does not appear to be a primary oncogenic driver, hFCRL1 could serve secondary roles in this setting. Especially intriguing is its upregulation as a function of lymphoma aggressiveness, which portends high-risk disease and an inferior clinical course. In conclusion, FCRL1 remains an understudied molecule in immunobiology. How and if hFCRL1 mechanistically contributes to the genesis or maintenance of lymphoproliferation and whether it can be targeted to improve clinical options for affected patients are obvious questions ripe for exploration.

## Author contributions

MM wrote the first draft of the manuscript. RD wrote and edited the manuscript. All authors contributed to the article and approved the submitted version.

## Glossary

**Table:**

Ab	Antibody
ABC	Activated B cell-like
AKT	Serine/threonine protein kinase B
BCR	B cell receptor
BITE	Bispecific T cell engager
BL	Burkitt’s lymphoma
BLNK	B cell linker protein (SLP-65)
c-Abl	Abelson murine leukemia virus tyrosine-protein kinase
CAR	Chimeric antigen receptor
CD	Cluster of differentiation
CFSE	Carboxyfluorescein diacetate succinimidyl ester
CLL	Chronic lymphocytic leukemia
DLBCL	Diffuse large B cell lymphoma
ERK	Extracellular signal-regulated kinase
FCRL	Fc receptor-like
GC	Germinal center
GCB	Germinal center B cell-like
GEP	Gene expression profiling
GRB2	Growth factor receptor-bound protein 2
Ig	Immunoglobulin
*IGHV*	Immunoglobulin heavy-chain variable region
ITAM	Immunoreceptor tyrosine-based activation motif
ITIM	Immunoreceptor tyrosine-based inhibitory motif
KLH	Keyhole limpet hemocyanin
mAb	Monoclonal antibody
MCL	Mantle cell lymphoma
MHG	Molecular high-grade
NF-κB	Nuclear factor kappa-light-chain-enhancer of activated B cells
NHL	Non-Hodgkin’s lymphoma
NP	4-hydroxy-3-nitrophenylacetyl
PC	Phosphorylcholine
PI3K	Phosphoinositide 3-kinase
SHIP1	Src homology 2 (SH2) domain containing inositol polyphosphate 5-phosphatase 1
shRNA	Short hairpin RNA
SOS1	Son of sevenless 1 guanine nucleotide exchange factor
SYK	Spleen-associated tyrosine kinase
TD	T cell-dependent
TI-2	T cell-independent type II
TIRFM	Total internal reflection fluorescence microscopy
TKI	Tyrosine kinase inhibitor
